# Potential Saving of Antibiotics for Respiratory Infections in Several European Countries: Insights from Market Research Data

**DOI:** 10.3390/antibiotics12071174

**Published:** 2023-07-11

**Authors:** André Gessner, Ludger Klimek, Ernest Kuchar, Ingrid Stelzmueller, Andrzej M. Fal, Peter Kardos

**Affiliations:** 1 Institute for Clinical Microbiology and Hygiene, University Clinic Regensburg, 93053 Regensburg, Germany; 2 Centre for Rhinology and Allergology, 65183 Wiesbaden, Germany; ludger.klimek@allergiezentrum.org; 3 Department of Pediatrics with Clinical Assessment Unit, Medical University of Warsaw, 02-091 Warsaw, Poland; ernest.kuchar@gmail.com; 4 Private Practice for Pulmonology, Internal Medicine and Pneumology, 5020 Salzburg, Austria; praxis@drstelzmueller.at; 5 Department of Allergy, Lung Diseases, and Internal Medicine, Central Clinical Hospital, Ministry of Interior, 02-507 Warsaw, Poland; amfal@wp.pl; 6 Lung Centre Frankfurt Maingau-Hospital, 60316 Frankfurt am Main, Germany; kardos@lungenpraxis-maingau.de

**Keywords:** market research, Europe, antimicrobial resistance, bronchitis, cough, pharyngitis, rhinosinusitis, antibiotics, ambulant, prescriptions

## Abstract

Antibiotics represent an essential pillar in the treatment of respiratory infections (RI). Overuse of antibiotics in avoidable cases and inappropriate application in bacterial infections facilitate treatment resistance, threatening their effectiveness and causing a significant healthcare challenge. We therefore assessed the savings potential for antibiotics in ambulant care of selected RI (bronchitis and cough, pharyngitis, rhinosinusitis) in several European countries based on market research data for the year 2019. Number of antibiotic packages sold in pharmacies varied, with highest values in Serbia and France, and lowest in Sweden and Switzerland. Selected RI contributed nearly half of overall ambulant antibiotic prescriptions, with around one fifth given for bronchitis and cough; the vast majority was estimated to be of viral origin with potentially avoidable antibiotic use. Antibiotic consumption for selected RI in eight European countries (Austria, Belgium, the Czech Republic, France, Germany, Poland, Slovakia, and Switzerland) amounted to nearly 100 million, with an overall savings potential between 66.2 and 83.7 million packages. The highest estimated volume of avoidable antibiotics was in France (44.7 million, 0.80 per capita), and lowest in Switzerland (1.4 million, 0.18 per capita). Due to substantial savings potential, prudent use of antibiotics and adequate application of alternatives should be promoted in daily practice.

## 1. Introduction

Antibiotics represent an essential pillar in the treatment of respiratory infections, specifically those caused or complicated by bacterial pathogens. Indeed, responsiveness of bacteria to antibiotics is vital for healthcare worldwide but is unfortunately increasingly compromised by antibiotic treatment resistance. Selection pressure and genetic mutations or acquisition of genetic material from already resistant bacteria facilitate the occurrence of resistance [1,2,3]. By 2015, the estimated burden of antibiotic-resistant infections caused by eight bacterial strains frequently encountered within the European Union and the European Economic Area (EU/EAA) was similar to that of influenza, tuberculosis, and HIV combined (reflected in cases, deaths, and disability-adjusted life-years). Tragically, it was associated with approximately 33,000 fatalities, as a median across the analyzed pathogens [4]. In addition, deaths assessed as attributable to the eight identified bacterial strains frequently encountered within the European Union and the European Economic Area had increased by a factor of 2.46 between 2007 and 2015. Third-generation-cephalosporin-resistant *Escherichia coli*, known to be associated with various infections including pneumonia [5,6], was heading the list with a four-fold increase in attributable deaths. Although antimicrobial resistance undoubtedly represents a worldwide issue, a systematic analysis of its global burden in 2019 found that sub-Saharan Africa and south Asia endured the highest burden of fatalities, while Australasia was the least affected [7]. It also highlighted that lower respiratory infections were the most burdensome infectious syndrome. Multiple drug resistance can further complicate matters by limiting treatment options and thus making adequate therapy particularly challenging [3,8]. As exposure to antibiotics is known to facilitate resistance [1,9,10], this data clearly illustrates the need for reducing antibiotic consumption by avoiding any unnecessary use in order to help tackle the issue of resistance and preserve the effectiveness of antibiotics. Indeed, as part of the global effort to address this healthcare challenge, corresponding guidelines and publicly available national action plans highlight its importance and detail strategies such as monitoring systems, raising awareness among relevant stakeholders, and advancing research efforts [11,12,13,14].

Since antibiotics are only effective against bacteria, they cannot treat infections of viral origin. Antibiotics have accordingly been described as having little to no benefit for common and mostly viral respiratory tract infections in otherwise healthy adults [15]. Analyzing more than 3.3 million episodes of respiratory tract infections, Petersen et al. concluded that antibiotics are not indicated for reducing the risk of serious complications in patients with respiratory tract infections, sore throat or otitis media [16]. A meta-analysis of relevant studies also found that antibiotics were not recommendable for the common cold [17]. Furthermore, recent experimental studies suggest that antibiotics can dampen antiviral immunity via microbiome perturbation [18]. Nevertheless, inappropriate antibiotic prescriptions for respiratory tract infections have been a long-standing issue [19,20]. Based on data from the 2018 Eurobarometer Report, cold and flu alone were responsible for one fifth of antibiotic consumption, with bronchitis and sore throat as the two most frequent indications [21]. Overall, this reinforces the debate around the excessive use of antibiotics for respiratory infections as an avoidable contributing factor for antibiotic resistance. In addition, overuse of antibiotics exposes patients to the risk of unnecessary side effects, including gastrointestinal complaints (e.g., nausea or diarrhea) and complications, such as *Clostridium difficile* infections or acute allergic reactions [22,23,24]. Various negative outcomes in later life, such as obesity, asthma, allergies or inflammatory bowel disease, can also occur due to interference with the natural gut microbiota vital for the maturation of the adaptive immune system [24]. Although valuable data on antibiotic consumption in different European countries has previously been reported [25,26,27], detailed estimates on the contribution of common respiratory infections and potential share of avoidable prescriptions in ambulatory care were yet to be provided. Our present work therefore, provides such estimates for three prevalent respiratory indications (bronchitis and cough, pharyngitis, rhinosinusitis) across several European countries, potentially beneficial for antimicrobial stewardship and future actions aimed at tackling the issue of excessive antibiotic consumption facilitating the development of resistance.

## 2. Results

**Figure 1:**
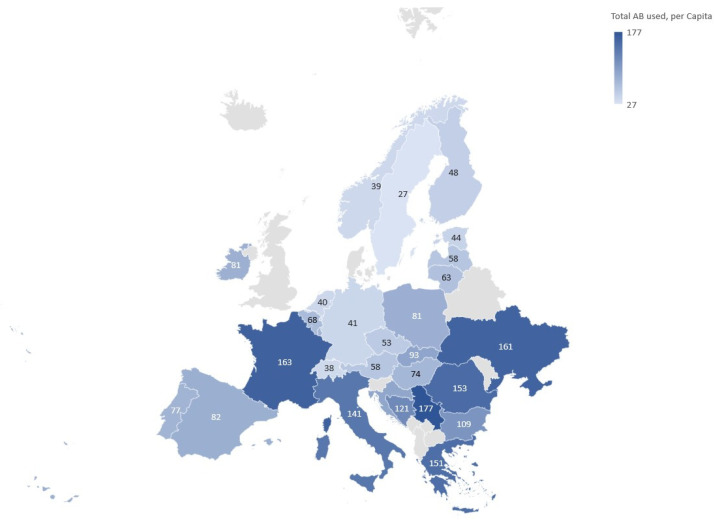
Antibiotic packages for ambulatory systemic treatment sold by pharmacies across Europe in 2019 based on data from [29]. Average value for the per capita index across 28 countries was set to 100, resulting in the displayed range of 27 to 177 total per capita antibiotic packages.

The number of packages sold for systemic antibiotic treatment in ambulatory care varied substantially across European pharmacies. Taking into account the population statistics for 2019 [28], highest values of packages per capita were determined for Serbia, France, and Ukraine (Figure 1). Countries on the lower side of the spectrum included Sweden, Switzerland, Norway, Netherlands, and Germany.

Eight countries had a total ambulant per capita consumption of antibiotics close to the overall value determined across 28 selected European countries (0.89 vs. 1.04). They were therefore deemed a suitable selection for further analysis (Table 1). Among these countries, by far the highest ambulant per capita consumption was found to be in France (index value of 163, Figure 1), and lowest in Switzerland (index 38), closely followed by Germany (index 41). Since ambulant care was mostly associated with higher antibiotic consumption (Table 1) and would likely offer a higher potential for reducing antibiotic consumption due to viral infections, our further analyses were focused on the ambulant rather than hospital setting.

Indications related to respiratory infections (ICD-10 codes, Appendix A), a suspected main driver of antibiotic consumption [21], were grouped into three categories for analysis: cough and bronchitis, pharyngitis, and rhinosinusitis. Based on prescription data for selected ICD-10 codes in France and Germany (representative of higher and lower antibiotic consumption, resp., Appendix B), an average proportion of prescriptions was estimated for the three indication categories. Taken together, these three categories were found to contribute nearly half of the overall ambulant antibiotic prescriptions (Table 2). Around one fifth of prescriptions were provided due to bronchitis and cough. A similar proportion was estimated for pharyngitis, whereas rhinosinusitis contributed less than half as much. Based on current knowledge described in pertinent literature [12,30,31,32,33,34,35,36,37,38,39,40,41,42], the vast majority of the infections were estimated to be of viral origin and thus contain potential for saving antibiotics, more apparently so for bronchitis and cough or rhinosinusitis compared to pharyngitis.

**Figure 2:**
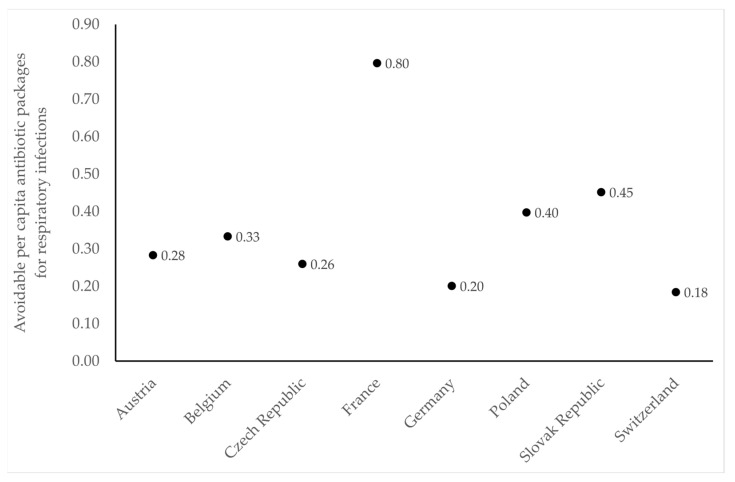
Avoidable per capita prescriptions of antibiotics for respiratory infections in ambulatory care based on antibiotic sales data and estimated share of viral infections presented in Table 2 as well as population statistics for 2019 [28].

Transferring the percentage margins of infections with viral origin determined for the three groups of respiratory indications to the eight selected European countries reveals a substantial savings potential regarding the use of antibiotics (Table 3). Indeed, maximal potential savings in individual countries would fall in the range of 1.4 million packages (in Switzerland) to 44.7 million packages (in France). Relative to total consumption across the selected countries, bronchitis and cough account for 17% to 19%, pharyngitis for 9.6% to 15.3%, and rhinosinusitis for 11.4% to 13%. The overall savings potential for antibiotics within these three groups of indications would thus fall in the range of 66.2 to 83.7 million packages and correspondingly account for up to 40.7% of prescriptions in ambulatory care. Translating these findings into avoidable antibiotic prescriptions per capita resulted in a range of 0.18 in Switzerland to 0.80 in France, with Slovakia approximately in the middle with a value of 0.45 (Figure 2).

**Figure 3:**
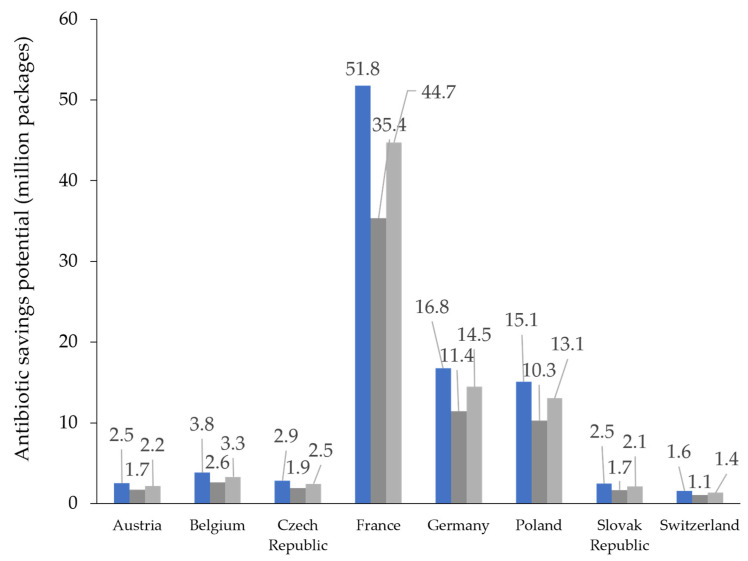
Antibiotic prescriptions for respiratory infections in ambulant care across individual countries (blue) and savings potential based on estimates for infections with viral origin (minimal saving, dark grey; maximal saving, light grey).

In the maximal as well as the minimal estimate based on data presented in Table 2 and Table 3, the overall savings potential for antibiotics assessed as prescribed for viral infections made up the majority of antibiotic packages sold in individual European countries (Figure 3).

**Figure 4:**
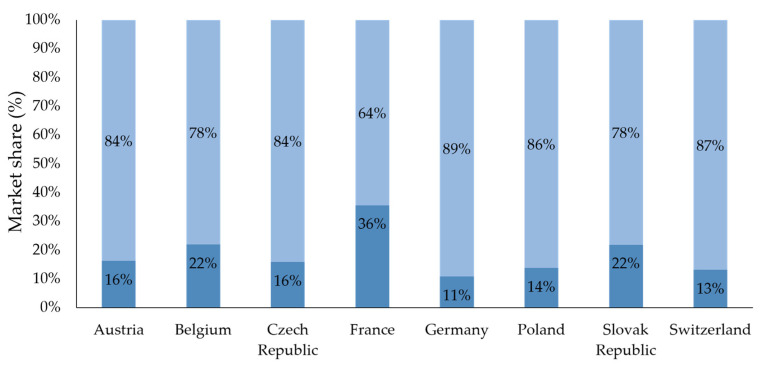
Estimated pharmacy sales of prescription (Rx) antibiotics (dark blue) versus over-the-counter (OTC) product sales (light blue) for the indication of pharyngitis based on data from IQVIA [44]. Bars show the proportionate distribution of products sold, with percentages indicating their market share.

Highlighted on the example of pharyngitis, countries with high volumes of antibiotic prescriptions showed a comparatively low volume of over-the-counter (OTC) products sold in pharmacies for corresponding treatment. In accordance with the above-reported data, lowest OTC sales in this category were found in France, with Germany and Switzerland at the higher side of the spectrum (Figure 4). Consistent with their market allocation, OTC-containing topical antibiotics were considered in this analysis. Their market share was mostly below 10% (Austria, 4.2%; Belgium, 1.3%; Switzerland, 6.7%; Germany, 7.1%), with the exception of Slovakia (12.8%).

Taken together, our estimates reveal a marked contribution of prevalent respiratory infections to antibiotic consumption across several European countries with substantial savings potential due to viral causes.

## 3. Discussion

Our analysis of market research data in Europe offers insights into the extent of antibiotic use for prevalent respiratory indications and corresponding potential for saving antibiotics. Due to its foundation in real-world data, this analysis reflects the magnitude of antibiotic prescriptions and thus provides an impression of their potential impact on a larger scale of several European countries. However, due to limited data availability, certain assumptions were made during the analysis, leading to limitations including the selection of ICD-10 codes in only two of the countries (Germany and France) as well as the classification of avoidable treatment, which might be subject to interpretation. Indeed, antibiotic treatment can also become necessary in viral infections, depending on individual circumstances (patient age, care setting) and the associated risk of potentially exacerbating existing conditions (e.g., COPD). Nevertheless, it is important to note that fear of complications, patient age and the setting of long-term aged care facilities have also previously been identified as factors facilitating inappropriate prescriptions [45,46,47,48,49]. In contrast, many mild bacterial infections can be adequately controlled by the immune system without the need for antibiotics [50,51]. These considerations are important for the assessment of avoidable antibiotic use but can only be undertaken with detailed information on individual cases, which would not have been available for the present work. Due to the scope of our work and the nature of the obtained data, antibiotics not purchased in pharmacies (e.g., obtained directly from physicians) could not be considered. In addition, it was not possible to account for any leftover antibiotics from previous years potentially used in 2019 or antibiotic packages only partially used in 2019 and thus leftover for potential future consumption. However, it is conceivable that these two categories would ultimately somewhat balance out, thus reducing their overall potential impact on antibiotic consumption. Our approach of assessing antibiotic prescriptions in the remaining countries based on an average for France and Germany is a rough estimate of approximate prescription volumes. In our analysis on pharyngitis, OTC antibiotics with active substances such as gramicidin, neomycin, bacitracin, and tyrothricin available for topical treatment of sore throat in some countries were considered in accordance with their market allocation. Altogether, we hope that sharing detailed information on the corresponding ICD-10 codes will provide an impression of varying prescription habits across European countries.

Due to the known correlation between exposure to antibiotics and development of antibiotic resistance, high consumption of antibiotics in ambulant care and the substantial contribution of respiratory indications found in our analysis offer important insights for daily practice. Although national action plans on antimicrobial resistance exist and community consumption of antibiotics has reduced in most European countries between 2019 and 2020, levels of resistance to critically important antibiotics in bacteria commonly responsible for healthcare-associated infections remained high (>20% to 50%) or very high (>50% to 70%) [11,25]. For instance, 2020 EU/EEA population-weighted mean percentages of resistance to third-generation cephalosporins in *Klebsiella pneumoniae* and to carbapenems in *Pseudomonas aeruginosa* and *Acinetobacter* species amounted to 34%, 18% and 38%, respectively [25]. Furthermore, consumption of systemic antibacterials within primary care in the EU/EAA area has remained relatively stable between 2020 and 2021, even showing increasing trends in some European countries, including France and Slovakia [52]. Our current estimates for the contribution of bronchitis and sore throat to antibiotic consumption are indeed also somewhat higher than those of the 2018 Eurobarometer Report [21], although it is important to note that in our analysis bronchitis is considered together with the indication of cough. Notwithstanding this difference in categorization, higher consumption would be consistent with the trend revealed by recent findings which indicate an increase in antibiotic consumption during the subsequent period of the COVID-19 pandemic [53,54,55]. In accordance with this trend, an increase in antibiotic resistance was also observed [56]. It is important to note that additional consideration of mild bacterial infections would expectedly further increase our estimates. It has previously been estimated that by the year 2050, deaths attributable to antimicrobial resistance would surpass those due to other individual causes, such as cancer or diabetes [57]. The need for urgent and targeted measures to reduce antibiotic consumption is thus apparent, particularly also in the field of respiratory infections. Apart from curbing the development of further resistance and reducing the burden of unnecessary side effects in patients, this could also have other positive implications for healthcare. For instance, decreased antibiotic consumption has been shown to coincide with a reduction in bacteremia caused by bacteria with respiratory transmission potential [58] and can correspondingly diminish the risk of further health issues in patients. Furthermore, drug resistance can even gradually decline during exposure to an antibiotic-free environment, with an antibiotic-specific extent of resistance loss, indicating a possible added benefit of restricting antimicrobial usage [59].

Some of the major factors identified as facilitating irrational use of antibiotics in Europe include lack of public knowledge and awareness, access to antibiotics without prescription and leftover antibiotics, inadequate medical training, and lack of rapid and sufficient diagnostic tests [60]. In Germany, general practitioners were found to be more likely to prescribe antibiotics for acute, non-complicated infections compared to other specialists [49]. Multiple measures are thus conceivable or recommendable due to previous successful use in reducing antibiotic consumption. Prescriber-focused interventions, including provision of clinical pathways and patient education materials, have been successful in appropriate reduction of antibiotic prescriptions for respiratory tract diagnoses [61,62]. Recommended measures for tackling the overuse of antibiotics in the treatment of outpatient acute respiratory infections also include eliminating antibiotic treatment of viral upper respiratory tract infections and bronchitis as well as improving adherence to prescribing guidelines for pharyngitis and sinusitis, along with improved access to virus diagnostic tests [46]. In addition, delayed prescriptions could be a safe and effective strategy to reduce antibiotic usage for acute respiratory infections [63,64,65]. Based on our estimates, an additional savings potential of up to 10%, and in the case of pharyngitis even up to 36%, could be unlocked if early-stage bacterial infections are treated with antibiotic-free alternatives. Where clinicians feel it would be safe not to prescribe antibiotics but rather advise a follow-up consultation in case of persisting respiratory symptoms, this approach would likely result in the least antibiotic use while maintaining similar patient satisfaction and clinical outcomes to delaying the prescription of antibiotics [63]. Finally, interventions that promote shared decision-making can at least lead to a short-term reduction of antibiotic prescriptions in primary care [66].

Antibiotics rarely benefit acute bronchitis, exacerbations of asthma and chronic bronchitis, acute pharyngitis and acute sinusitis, although they are commonly prescribed in such cases [15]. Complex factors underlying the overprescribing of antibiotics need to be investigated and thoroughly understood. Due to the impact of patient expectations on prescribing behavior, practitioners should be more proactive in explaining to their patients that antibiotics will not help to relieve their symptoms, which will often respond to other medication [15]. Indeed, administering phytopharmaceuticals has been shown to correlate with a lower need for antibiotic prescriptions and shorter sick leaves due to acute respiratory infections [67]. Awareness should accordingly be raised for symptomatic treatment options e.g., the antiseptic agent octenidine for sore throat and the anti-inflammatory and mucolytic cineole applicable in various respiratory conditions, including rhinosinusitis and acute bronchitis [68,69,70]. Improving consultations to effectively involve patients in reaching and owning evidence-based prescribing decisions will be important for improving the management of respiratory tract infections in primary care [48]. In addition, national-level interventions to reduce inappropriate demand and access to antibiotics and antimicrobial stewardship programs can be effective in facilitating rational use and reducing antibiotic consumption [20,71,72,73]. Involving all stakeholders will be essential for necessary progress in healthcare practices.

## 4. Materials and Methods

Data on antibiotic packages sold by pharmacies within ambulant care in Europe were obtained under license from IQVIA’s IQVIA MIDAS^®^ information service for the year 2019 (copyright by IQVIA; all rights reserved) [29]. To our knowledge, this time period precedes registered cases of COVID-19 in Europe, which undoubtedly had a substantial impact on the transmission of respiratory infections and antibiotic consumption. In-depth analysis regarding causes for antibiotic consumption based on the International Statistical Classification of Diseases and Related Health Problems (ICD-10) codes [74] was focused on Austria, Belgium, the Czech Republic, France, Germany, Poland, Slovakia, and Switzerland. The color-coded map of Europe presented in Figure 1 was created using GeoNames. Data obtained for prescriptions in Germany and France [43] were considered adequate representatives of the lower and higher side of the spectrum and their average was thus used to derive possible distribution of antibiotic consumption across selected indications in each of the eight countries entering into the analysis. ICD-10 codes related to rhinosinusitis, pharyngitis, and bronchitis and cough (Appendix A) were selected for analysis from a pool of available data in the two representative countries based on relevance for infections likely to be of viral origin. The potential for saving antibiotics was estimated based on medical justification of prescriptions. Prescriptions were considered justified in cases of escalated bacterial infections, viral infections with bacterial superinfections (or corresponding risk), as well as any other medical condition warranting the use of antibiotics. Antibiotic prescriptions for viral respiratory infections were considered avoidable (non-justified).

Market share of OTC remedies [44] vs. prescription antibiotics was highlighted using the example of pharyngitis. Indications contained under the term pharyngitis are particularly well suited for analysis, as they are often treated with prescription antibiotics but, when treated with OTC products, enable clear distinction between products used specifically for their treatment as opposed to other related indications. Total antibiotic consumption for pharyngitis was determined as the sum of IQVIA OTC Segment 01C1 Sore Throat Remedies unit consumption and the estimated share of antibiotics prescribed for pharyngitis, accounting for the total annual consumption across individual countries. Where appropriate, mean values were generated and are reported along with standard deviation. The statements, findings, conclusions, views, and opinions contained and expressed in this article are based in part on data obtained under license from IQVIA Ltd. and/or its affiliated or subsidiary entities (“IQVIA”). The statements, findings, conclusions, views, and opinions contained and expressed herein are not necessarily those of IQVIA.

## 5. Conclusions

Nearly half of the overall antibiotic consumption in the ambulant sector across various European countries is attributable to respiratory infections. Due to the predominantly viral origin of respiratory infections, a substantial proportion of corresponding antibiotic prescriptions is avoidable. Since exposure to antibiotics facilitates treatment resistance and can be associated with side effects, appropriate measures should be undertaken to improve healthcare practices by preventing unnecessary use of antibiotics. In this regard, it will be important to consider the impact of the COVID-19 pandemic on antibiotic consumption and antimicrobial resistance and evaluate corresponding trends prior to and following the period of the pandemic in order to optimize antibiotic stewardship efforts. Some of the measures that could facilitate improvements in daily practice might include medical training on antibiotic prescribing, shared decision-making and potential for safe delayed prescriptions, patient education on appropriate use and risks associated with antibiotic consumption and improving diagnostic tools. Corresponding strategies should contain a multifaceted approach to reach prescribers and patients on pertinent issues and also raise awareness on alternative, symptomatic treatments known to be suitable for meeting the needs of patients and thus beneficial for curbing the development of antibiotic resistance.

## Figures and Tables

**Table 1:** Antibiotic sales volumes based on data from IQVIA [29] and population statistics [28] for eight selected European countries.

Country	Total Antibiotic Sales (Million Packages)	Antibiotic Sales in the Ambulant Setting (Million Packages)	Antibiotic Sales in the Hospital Setting (Million Packages)	Population (Million)
Austria	7.6	5.4	2.3	8.9
Belgium	24.9	8.1	16.7	11.5
Czech Republic	9.7	6.1	3.7	11
France	186.5	110.1	76.5	65
Germany	188.4	35.6	152.8	83.2
Poland	48.9	32.1	16.8	38
Slovakia	6.1	5.2	0.8	5.5
Switzerland	5.4	3.4	2	8.6
Total	477.6	206.0	271.6	231.6

**Table 2:** Contribution of various respiratory indications to ambulant antibiotic prescriptions (average for Germany and France) based on sales and prescription data [29,43], with estimated causes of infections based on pertinent literature.

	Estimated Average Prescription Volumes Based on Values for Germany and France		
	Bronchitis and Cough	Pharyngitis	Rhinosinusitis
Ambulant antibiotic prescriptions (mean % ± SEM)	20.02 ± 0.37	19.13 ± 3.25	7.92 ± 2.42
	Origin of respiratory infections based on pertinent literature		
Cause of infection (%)	Estimates based on [12,30,33,34,35]	Estimates based on [32,36,37,38]	Estimates based on [31,39,40,41,42]
Viral	85–95	50–80	70–80
Bacterial	1–10	5–36	0.5–2
Non-viral, other	5–10	10	20

**Table 3:** Estimated antibiotic prescriptions for ambulant treatment per indication based on prescription volumes for respiratory indications in Germany and France (as presented in Table 2 and indicated here below each category). Savings potential is based on estimated proportions of infections with viral origin presented in Table 2.

Country	Total Ambulant Antibiotic Packages (Million Packages) [29]	Bronchitis and Cough (20.02%)	Pharyngitis (19.13%)	Rhinosinusitis (7.92%)	Respiratory Infections, Ambulant, Subtotal (47.06%)	Other Indications, Ambulant, Subtotal (52.94%)
Austria	5.4	1.1	1	0.4	2.5	2.8
Belgium	8.1	1.6	1.6	0.6	3.8	4.3
Czech Republic	6.1	1.2	1.2	0.5	2.9	3.2
France	110.1	22	21.1	8.7	51.8	58.3
Germany	35.6	7.1	6.8	2.8	16.8	18.8
Poland	32.1	6.4	6.1	2.5	15.1	17
Slovakia	5.2	1	1	0.4	2.5	2.8
Switzerland	3.4	0.7	0.6	0.3	1.6	1.8
Total	206	41.2	39.4	16.3	96.9	109
	Savings potential due to viral cause					
Antibiotic packages (million)	n. a.	35–39.2	19.7–31.5	11.4–13	66.2–83.7	n. a.
% of total consumption		17–19%	9.6–15.3%	5.5–6.3%	32.1–40.7%	

## Data Availability

All relevant data are reported within this article and its Appendix A and Appendix B.

## Appendix A

**Table A1:** Overview of the ICD-10 codes considered within the analysis.

	Rhinosinusitis	Pharyngitis	Bronchitis and Cough
ICD-10 codes	J01 ACUTE SINUSITIS	J00 AC NASOPHARYNGITIS	J15 BACTERIAL PNEUMONIA NEC
	J01.0 AC MAXILLARY SINUSITIS	J00.0 AC NASOPHARYNGITIS	J15.0 PNEUM-KLEBSIELL PNEUME
	J01.1 ACUTE FRONTAL SINUSITIS	J02 ACUTE PHARYNGITIS	J15.1 PNEUMONIA-PSEUDOMONAS
	J01.2 AC ETHMOIDAL SINUSITIS	J02.0 STREP PHARYNGITIS	J15.2 PNEUMONIA-STAPH
	J01.3 AC SPHENOIDAL SINUSITIS	J02.8 AC PHARYNG-O/SPEC ORGISM	J15.3 PNEUM-STREP GROUP B
	J01.4 ACUTE PANSINUSITIS	J02.9 ACUTE PHARYNGITIS UNSP	J15.4 PNEUM-OTH STREPTOCOCCI
	J01.8 OTHER ACUTE SINUSITIS	J03 ACUTE TONSILLITIS	J15.6 PNEUM-O/GRAM NEG BACT
	J01.9 ACUTE SINUSITS UNSP	J03.0 STREP TONSILLITIS	J15.7 PNEUM-MYCOPLASMA PNEUM
	J06 AC URTI-MULT/UNSP STS	J03.8 AC TONSILL-O/SPEC ORGS	J15.8 OTHER BACT PNEUMONIA
	J06.0 ACUTE LARYNGOPHARYNGITIS	J03.9 ACUTE TONSILLITIS UNSP	J15.9 BACT PNEUMONIA UNSP
	J06.8 O/AC UP RESP INF MULT ST	J04 AC LARYNGITIS/TRACHEITIS	J18 PNEUMONIA ORGISM UNSP
	J06.9 AC UPP RESP INFECT UNSP	J04.0 ACUTE LARYNGITIS	J18.0 BRONCHOPNEUMONIA UNSP
	J31 CHR RHIN NASO/PHARYNG	J04.1 ACUTE TRACHEITIS	J18.1 LOBAR PNEUMONIA UNSP
	J31.0 CHRONIC RHINITIS	J04.2 ACUTE LARYNGOTRACHEITIS	J18.2 HYPOSTATIC PNEUM UNSP
	J31.1 CHRONIC NASOPHARYNGITIS	J35 CHR DIS-TONSILS/ADENOIDS	J18.8 OTH PNEUM ORGANISM UNSP
	J31.2 CHRONIC PHARYNGITIS	J35.0 CHRONIC TONSILLITIS	J18.9 PNEUMONIA UNSPECIFIED
		J35.1 HYPERTROPHY OF TONSILS	J20 ACUTE BRONCHITIS
		J35.2 HYPERTROPHY OF ADENOIOS	J20.0 AC BRONCH-M PNEUMONIAE
		J35.3 HYPERTROPHY TONS + ADEN	J20.1 AC BRONCH-H INFLUENZAE
		J35.8 O/CHR DIS TONSIL/ADENOID	J20.2 AC BRONCHITIS-STREP
		J35.9 CHR DIS TONS/ADEN UNSP	J20.4 AC BRONCH-PARAINFLU VIR
		R07 PAIN IN THROAT AND CHEST	J20.5 AC BRONCH-RESP SYNCY VIR
		R07.0 PAIN IN THROAT	J20.6 AC BRONCHITIS-RHINOVIR
		R07.1 CHEST PAIN ON BREATHING	J20.8 AC BRONCH-O/SPEC ORGISMS
		R07.2 PRECORDIAL PAIN	J20.9 ACUTE BRONCHITIS UNSP
		R07.3 OTHER CHEST PAIN	J21 ACUTE BRONCHIOLITIS
		R07.4 CHEST PAIN UNSPECIFIED	J21.0 AC BRON-RESP SYNCY VIR
			J21.1 AC BRONCH METAPNEUMOVIR
			J21.8 AC BRONCHIOL-O/SPEC ORG
			J21.9 AC BRONCHIOLITIS UNSP
			J22 UNSP AC LOW RESP INFECT
			J22.0 UNSP AC LOW RESP INFECT
			J40 BRONCH NOT SPEC AC/CHR
			J40.0 BRONCH NOT SPEC AC/CHR
			J42 UNSP CHRONIC BRONCHITIS
			J42.0 UNSP CHRONIC BRONCHITIS
			J68 RESP COND-CHEM/GAS ETC
			J68.0 BRONC/PNEUM-CHEM GAS ETC
			J68.2 URT INFL-CHEM GAS ETC
			R05 COUGH
			R05.0 COUGH

Following related ICD-10 codes were not included in the analysis: J33 Polyp, J13 Pneumococci, J09 and J10 for Influenza due to their specific therapy needs; ICD-10 codes related to allergic reactions, as they are out of the scope of the present analysis with focus on respiratory infections (J30 Allergic rhinosinusitis and J43–48 for Asthma).

## Appendix B

**Table A2:** Detailed overview of the annual prescriptions from 2019 in Germany and France for selected ICD-10 codes based on [43] used to derive average prescriptions for the indication groups presented in Table 2.

Indication (ICD-10)	France Packages	France (%)	Germany Packages	Germany (%)
Total	30,496,864	100.00%	28,179,583	100.00%
Selected RI total (average 47.06%)	14,487,296	47.50%	13,127,509	46.59%
Other indication total (average 52.94%)	16,009,568	52.50%	15,052,074	53.41%
Bronchitis and Cough average 20.02% (SEM 0.37%)	5,996,236	19.66%	5,749,440	20.40%
J15 BACTERIAL PNEUMONIA NEC	9192	0.03%	35,426	0.13%
J15.0 PNEUM-KLEBSIELL PNEUME		0.00%	0	0.00%
J15.1 PNEUMONIA-PSEUDOMONAS		0.00%	245	0.00%
J15.2 PNEUMONIA-STAPH	0	0.00%	0	0.00%
J15.3 PNEUM-STREP GROUP B		0.00%	66	0.00%
J15.4 PNEUM-OTH STREPTOCOCCI		0.00%	406	0.00%
J15.6 PNEUM-O/GRAM NEG BACT	0	0.00%		0.00%
J15.7 PNEUM-MYCOPLASMA PNEUM	1300	0.00%	10,601	0.04%
J15.8 OTHER BACT PNEUMONIA		0.00%	9329	0.03%
J15.9 BACT PNEUMONIA UNSP	7892	0.03%	14,779	0.05%
J18 PNEUMONIA ORGISM UNSP	347,804	1.14%	657,987	2.33%
J18.0 BRONCHOPNEUMONIA UNSP	82,692	0.27%	259,214	0.92%
J18.1 LOBAR PNEUMONIA UNSP		0.00%	12,608	0.04%
J18.2 HYPOSTATIC PNEUM UNSP		0.00%	1501	0.01%
J18.8 OTH PNEUM ORGANISM UNSP		0.00%	8391	0.03%
J18.9 PNEUMONIA UNSPECIFIED	265,112	0.87%	376,273	1.34%
J20 ACUTE BRONCHITIS	1,892,192	6.20%	2,813,329	9.98%
J20.0 AC BRONCH-M PNEUMONIAE		0.00%	3239	0.01%
J20.1 AC BRONCH-H INFLUENZAE		0.00%	390	0.00%
J20.2 AC BRONCHITIS-STREP		0.00%	3327	0.01%
J20.4 AC BRONCH-PARAINFLU VIR		0.00%	346	0.00%
J20.5 AC BRONCH-RESP SYNCY VIR		0.00%	98	0.00%
J20.6 AC BRONCHITIS-RHINOVIR	3900	0.01%	4687	0.02%
J20.8 AC BRONCH-O/SPEC ORGISMS	0	0.00%	196,145	0.70%
J20.9 ACUTE BRONCHITIS UNSP	1,888,292	6.19%	2,605,097	9.24%
J21 ACUTE BRONCHIOLITIS	55,860	0.18%	9394	0.03%
J21.0 AC BRON-RESP SYNCY VIR		0.00%	343	0.00%
J21.1 AC BRONCH METAPNEUMOVIR		0.00%	0	0.00%
J21.8 AC BRONCHIOL-O/SPEC ORG	0	0.00%	756	0.00%
J21.9 AC BRONCHIOLITIS UNSP	55,860	0.18%	8295	0.03%
J22 UNSP AC LOW RESP INFECT		0.00%	136,613	0.48%
J22.0 UNSP AC LOW RESP INFECT		0.00%	136,613	0.48%
J40 BRONCH NOT SPEC AC/CHR	2,223,900	7.29%	1,895,107	6.73%
J40.0 BRONCH NOT SPEC AC/CHR	2,223,900	7.29%	1,895,107	6.73%
J42 UNSP CHRONIC BRONCHITIS	48,956	0.16%	88,704	0.31%
J42.0 UNSP CHRONIC BRONCHITIS	48,956	0.16%	88,704	0.31%
J68 RESP COND-CHEM/GAS ETC	748	0.00%	201	0.00%
J68.0 BRONC/PNEUM-CHEM GAS ETC	276	0.00%	201	0.00%
J68.2 URT INFL-CHEM GAS ETC	472	0.00%	0	0.00%
R05 COUGH	1,417,584	4.65%	112,679	0.40%
R05.0 COUGH	1,417,584	4.65%	112,679	0.40%
Rhinosinusitis average 7.92% (SEM 2.42%)	1,705,860	5.59%	2,939,290	10.43%
J01 ACUTE SINUSITIS	1,537,896	5.04%	949,587	3.37%
J01.0 AC MAXILLARY SINUSITIS	2836	0.01%	332,049	1.18%
J01.1 ACUTE FRONTAL SINUSITIS	456	0.00%	94,028	0.33%
J01.2 AC ETHMOIDAL SINUSITIS	15,424	0.05%	5886	0.02%
J01.3 AC SPHENOIDAL SINUSITIS	5136	0.02%	9743	0.03%
J01.4 ACUTE PANSINUSITIS	0	0.00%	56,790	0.20%
J01.8 OTHER ACUTE SINUSITIS	3332	0.01%	37,563	0.13%
J01.9 ACUTE SINUSITIS UNSP	1,510,712	4.95%	413,528	1.47%
J06 AC URTI-MULT/UNSP STS	90,968	0.30%	1,959,802	6.95%
J06.0 ACUTE LARYNGOPHARYNGITIS	0	0.00%	144,004	0.51%
J06.8 O/AC UP RESP INF MULT ST	44,380	0.15%	199,339	0.71%
J06.9 AC UPP RESP INFECT UNSP	46,588	0.15%	1,616,459	5.74%
J31 CHR RHIN NASO/PHARYNG	76,996	0.25%	29,901	0.11%
J31.0 CHRONIC RHINITIS	73,644	0.24%	27,568	0.10%
J31.1 CHRONIC NASOPHARYNGITIS	1596	0.01%	337	0.00%
J31.2 CHRONIC PHARYNGITIS	1756	0.01%	1996	0.01%
Pharyngitis (Sore Throat) Average 19.13% (SEM 3.25%)	6,785,200	22.25%	4,438,779	15.75%
J00 AC NASOPHARYNGITIS	1,968,860	6.46%	149,187	0.53%
J00.0 AC NASOPHARYNGITIS	1,968,860	6.46%	149,187	0.53%
J02 ACUTE PHARYNGITIS	3,160,696	10.36%	1,122,357	3.98%
J02.0 STREP PHARYNGITIS	52,980	0.17%	43,735	0.16%
J02.8 AC PHARYNG-O/SPEC ORGISM	5208	0.02%	39,537	0.14%
J02.9 ACUTE PHARYNGITIS UNSP	3,102,508	10.17%	1,039,085	3.69%
J03 ACUTE TONSILLITIS	98,840	0.32%	2,626,821	9.32%
J03.0 STREP TONSILLITIS	728	0.00%	349,495	1.24%
J03.8 AC TONSILL-O/SPEC ORGS		0.00%	80,982	0.29%
J03.9 ACUTE TONSILLITIS UNSP	98,112	0.32%	2,196,344	7.79%
J04 AC LARYNGITIS/TRACHEITIS	1,415,248	4.64%	472,806	1.68%
J04.0 ACUTE LARYNGITIS	302,504	0.99%	337,906	1.20%
J04.1 ACUTE TRACHEITIS	904,620	2.97%	35,817	0.13%
J04.2 ACUTE LARYNGOTRACHEITIS	208,124	0.68%	99,083	0.35%
J35 CHR DIS-TONSILS/ADENOIDS	3868	0.01%	36,505	0.13%
J35.0 CHRONIC TONSILLITIS	3176	0.01%	23,895	0.08%
J35.1 HYPERTROPHY OF TONSILS	284	0.00%	5629	0.02%
J35.2 HYPERTROPHY OF ADENOIDS	408	0.00%	5296	0.02%
J35.3 HYPERTROPHY TONS + ADEN	0	0.00%	0	0.00%
J35.8 O/CHR DIS TONSIL/ADENOID	0	0.00%	916	0.00%
J35.9 CHR DIS TONS/ADEN UNSP		0.00%	769	0.00%
R07 PAIN IN THROAT AND CHEST	137,688	0.45%	31,103	0.11%
R07.0 PAIN IN THROAT	121,740	0.40%	29,770	0.11%
R07.1 CHEST PAIN ON BREATHING	808	0.00%	0	0.00%
R07.2 PRECORDIAL PAIN	1168	0.00%		0.00%
R07.3 OTHER CHEST PAIN	3004	0.01%	0	0.00%
R07.4 CHEST PAIN UNSPECIFIED	10,968	0.04%	1333	0.00%
